# Expression analysis of porcine miR-33a/b in liver, adipose tissue and muscle and its potential role in fatty acid metabolism

**DOI:** 10.1371/journal.pone.0245858

**Published:** 2021-01-26

**Authors:** Lourdes Criado-Mesas, Maria Ballester, Daniel Crespo-Piazuelo, Magí Passols, Anna Castelló, Armand Sánchez, Josep M. Folch

**Affiliations:** 1 Plant and Animal Genomics, Centre for Research in Agricultural Genomics (CRAG), CSIC-IRTA-UAB-UB, Campus UAB, Bellaterra, Spain; 2 Animal Breeding and Genetics Programme, Institute for Research and Technology in Food and Agriculture (IRTA), Torre Marimon, Caldes de Montbui, Spain; 3 Departament de Ciència Animal i dels Aliments, Facultat de Veterinària, Universitat Autònoma de Barcelona, Bellaterra, Spain; 4 Teagasc, Pig Development Department, Animal & Grassland Research & Innovation Centre, Moorepark, Fermoy, Ireland; INIA, SPAIN

## Abstract

*mir-33a* and *mir-33b* are co-transcribed with the *SREBF2* and *SREBF1* transcription factors, respectively. The main role of *SREBF1* is the regulation of genes involved in fatty acid metabolism, while *SREBF2* regulates genes participating in cholesterol biosynthesis and uptake. Our objective was to study the expression of both miR-33a and miR-33b, together with their host *SREBF* genes, in liver, adipose tissue and muscle to better understand the role of miR-33a/b in the lipid metabolism of pigs. In our study, the expression of miR-33a, miR-33b and *SREBF2* in liver, adipose tissue, and muscle was studied in 42 BC1_LD (25% Iberian x 75% Landrace backcross) pigs by RT-qPCR. In addition, the expression of *in-silico* predicted target genes and fatty acid composition traits were correlated with the miR-33a/b expression. We observed different tissue expression patterns for both miRNAs. In adipose tissue and muscle a high correlation between miR-33a and miR-33b expression was found, whereas a lower correlation was observed in liver. The expression analysis of *in-silico* predicted target-lipid related genes showed negative correlations between miR-33b and *CPT1A* expression in liver. Conversely, positive correlations between miR-33a and *PPARGC1A* and *USF1* gene expression in liver were observed. Lastly, positive and negative correlations between miR-33a/b expression and saturated fatty acid (SFA) and polyunsaturated fatty acid (PUFA) content, respectively, were identified. Overall, our results suggested that both miRNAs are differentially regulated and have distinct functions in liver, in contrast to muscle and adipose tissue. Furthermore, the correlations between miR-33a/b expression both with the expression of *in-silico* predicted target-lipid related genes and with fatty acid composition, opens new avenues to explore the role of miR33a/b in the regulation of lipid metabolism.

## Introduction

Pork is one of the most consumed meats in the world, being meat quality a relevant trait for both the meat industry and consumers. Among meat quality characteristics, intramuscular fat (IMF) content and fatty acid (FA) composition determine not only meat flavour, tenderness, firmness and juiciness, but also the healthiness of the product [1, 2]. In addition, the pig is considered a good animal model for biomedical research because of its similarities with humans, and has been used to identify drug targets against human diseases, such as obesity [3].

Liver, adipose tissue and skeletal muscle are the principal metabolic organs involved in the regulation of lipid metabolism and, therefore, play an important role in the determination of IMF content and FA composition. In pigs, liver participates in the synthesis and secretion of very low-density proteins, *de novo* cholesterol synthesis and fatty acid β-oxidation. In addition, liver and adipose tissue are involved in de novo fatty acid synthesis [4], with a higher contribution from adipose tissue. Furthermore, adipose tissue is an organ acting in lipid storage and maintenance of metabolic homeostasis, being the major source of circulating free FAs [5, 6]. Muscle is an important site for glucose uptake and storage, and a reservoir of amino acids, used for protein synthesis and energy production [7]. The lipid metabolism pathways are cross-regulated among liver, adipose tissue and muscle, and have been extensively studied.

Besides the transcriptional gene expression regulation, microRNAs (miRNAs) have emerged as important post-transcriptional regulators of the genes involved in lipid metabolism in different porcine tissues [8]. miRNAs are small RNA molecules that prevent the production of proteins or degrade the mRNA [9]. They play important roles in diverse regulatory pathways of many cellular processes and diseases. Members of the miR-33 family, which includes *mir-33a* and *mir-33b*, are located in *sterol regulatory element binding transcription factor 2* (*SREBF2*) intron 13 and *SREBF1* intron 16, respectively, and were reported to be co-transcribed with their host genes. SREBP transcription factors are well-known master regulators of lipid homeostasis. *SREBF1* regulates genes mainly involved in fatty acid metabolism, while *SREBF2* regulates genes involved in cholesterol biosynthesis and uptake [10, 11]. Pig miR-33a/b sequences differ only in three nucleotides, have the same seed sequence, and are conserved with the human homologous genes. In line with the regulatory functions of their host genes, human miR-33b was reported to regulate the insulin signalling pathway and glucose synthesis, which affected gluconeogenesis pathways [12, 13], and miR-33a was involved in the regulation of genes of cholesterol synthesis [14, 15]. In pigs, only miR-33b has been reported to play an important role in adipogenesis and lipogenesis in adipose tissue [16].

The aim of this work was to study the expression of miR-33a and miR-33b, together with their host *SREBF* genes, in the three main metabolic tissues, liver, adipose tissue and muscle, and the effect of both miR-33 genes on FA composition measured in muscle and adipose tissue, to better understand their role in lipid metabolism in swine.

## Methods

### Ethics statement

Animal care and procedures were performed following national and institutional guidelines for the Good Experimental Practices and approved by the Ethical Committee of the Institution (IRTA- Institut de Recerca i Tecnologia Agroalimentàries).

### Animal samples

The animal material used in this study comes from the IBMAP experimental cross population, which was generated by crossing three Iberian (Guadyerbas line) boars with 31 Landrace sows. Five F_1_ males were backcrossed with 25 Landrace sows thereafter (BC1_LD) [17]. In the current work, we randomly selected 42 pigs from the BC1_LD (25% Iberian x 75% Landrace) generation, being 6 males and 36 females. All animals were fed *ad libitum* with a cereal-based commercial diet and maintained under intensive conditions. The average age at slaughter was 174.5 days and the average weight at slaughter was 96.7 kg. After slaughter, liver, adipose tissue and *Longissimus dorsi* muscle samples were collected and immediately snap-frozen in liquid nitrogen and stored at -80°C until analysis.

### Phenotypic data

Composition of FAs with 12–22 carbons was determined in muscle [18] and backfat adipose tissue [19] using a protocol based on gas chromatography of methyl esters [17]. The percentage of the content of each FA was calculated afterwards in addition to the overall percentage of saturated FAs (SFA), monounsaturated FAs (MUFA) and polyunsaturated FAs (PUFA). Descriptive statistics of intramuscular fat and backfat FA composition and FA indices are presented in the S1 Table.

### Reverse transcription quantitative PCR (RT-qPCR)

A total of 42 pigs were used for the gene expression studies. Total RNA was purified from 50 mg of liver or from 150 mg of adipose tissue directly homogenising the samples in 1 mL of TRIzol Reagent with a polytron device. In the case of muscle (*Longissimus dorsi*) samples, 100 mg were submerged in liquid nitrogen and ground with a mortar and a pestle before adding 1 mL of TRIzol. For the miRNA expression assay, 200 μL of chloroform were added and samples were centrifuged to separate the nucleic acids and proteins from the RNA. Supernatant was collected to a new tube and total RNA was precipitated by adding 500 μL of isopropanol and washed with 75% ethanol [20]. For the mRNA expression assay, total RNA was obtained using the RiboPure kit (Ambion), following the producer’s recommendations. In both cases the RNA was resuspended with 100 μL in liver samples and 50 μL in adipose tissue and muscle samples with RNAse free water. RNA concentration and purity was measured using a NanoDrop ND-1000 spectrophotometer (NanoDrop products) and RNA integrity was checked by using an Agilent Bioanalyzer-2100 (Agilent Technologies).

For the miRNA expression assay, total RNA was reverse transcribed into cDNA with the Taqman Advanced miRNA cDNA synthesis kit (Applied Biosystems) by using 2 μL (5 ng/μL) of total RNA in a final reaction volume of 30 μL. Then, 5 μL of the resulting RT reactions were amplified in a final volume of 50 μL following manufacturer's instructions, in order to increase uniformly the amount of cDNA for all miRNAs. Finally, pre-amplified cDNA was diluted 1/10 for RT-qPCR. A negative control was made for each tissue with no reverse transcriptase added. cDNA was stored at -20°C until use. For the mRNA expression assay, 1 μg of total RNA was reverse-transcribed into cDNA in 20 μL reactions using random hexamer primers and the High-Capacity cDNA Reverse Transcription kit (Applied Biosystems), following the manufacturer’s instructions.

Pre-designed Taqman MicroRNA Assays (Applied Biosystems) were used for hsa-miR-33a-5p (reference 478347), hsa-miR-33b-5p (reference 478479), hsa-miR-let7a-5p (reference 478575) and hsa-miR-26a-5p (reference 477995). Primers were designed for *SREBF2* gene and reported in S2 Table. Standard curves were made with serial dilutions from a pool of cDNA to evaluate the performance of our RT-qPCR assays, and high PCR efficiencies were obtained. Relative quantification of hsa-miR-33a, hsa-miR-33b and SREBF2 by RT-qPCR was performed in a QuantStudioTM 12K Flex Real-Time PCR System (ThermoFisher Scientific) using a 384-well plate and all reactions were done per triplicate. To quantify the miRNAs, a final volume reaction of 15 μL containing 1x Taqman Fast Advanced master mix (Applied Biosystems), 1x Taqman Advanced miRNA Assay (Applied Biosystems) and 3.75 μL of pre-amplified cDNA diluted 1/10 was used. To quantify the *SERBF2* expression, also a final volume reaction of 15 μL containing 1x SybrSelect master mix (Applied Biosystems), 300nM primers and 3.75 μL of cDNA diluted ½ was used5. miR-let7a and miR-26a were used as porcine reference miRNAs and were chosen according to the bibliography [21, 22], and *ACTB* and *TBP* were used as porcine reference mRNAs (mRNA primers are reported in S2 Table) [23–25]. The PCR thermal cycle was: 2 min at 50°C, 10 min at 95°C, 40 cycles of 15 sec at 95°C and 1 min at 60°C. Moreover, a melting profile (95°C for 15 sec, 60°C for 15 sec and a gradual increase in temperature with a ramp rate of 1% up to 95°C) was added following the thermal cycling protocol, to assess for the specificity of the reactions. Data was analysed with the ThermoFisher Cloud software 1.0 (Applied Biosystems), PCR efficiencies were between 94.44 and 106.19% and the 2^-ΔCt^ [26] method was applied (S3 Table). *SREBF1* mRNA expression data in liver, adipose tissue, and muscle was previously generated by Ballester *et al*. 2017, Revilla *et al*. 2018 and Puig-Oliveras *et al*. 2016, respectively.

### miRNA target genes

Previously published studies of our group studied the expression of 84 lipid-related genes in liver, adipose tissue and/or muscle [23–25]. In these works, gene expression was quantified by qPCR in a set of animals which included the 42 animals of the present work [23–25]. The complete list of genes and the tissue where each gene expression was analysed is summarized in S4 Table.

Porcine mRNA 3’UTRs sequences were downloaded from the Ensembl database and Seqkit tool [27] was used to search by homology those mRNA 3’UTR sequences matching with 7mer seed miRNA sequence. Afterwards, only genes with a miRNA-33a/b binding site in their 3’UTR were considered for gene expression correlation analysis. Additionally, we assessed the conservation and confidence of the miR-33a/b putative target sites among other mammal species by using the TargetScan webserver [28].

### Statistical analysis

Normalization of data was checked using the Shapiro-Wilk test in R [29] and log_2_ transformation of the 2^-ΔCt^ value was applied if necessary. Means were compared using Tukey Honest Significant Difference (HSD) test [30]. Pearson’s correlations were performed among target gene expression or FA composition and miR-33a/b quantification using R software. The FDR (False Discovery Rate) method of Benjamini and Hochberg [31] was applied for the correction of multiple tests using the *p*.*adjust* function of R software.

## Results

### miR-33a and miR-33b expression in liver, adipose tissue and muscle

**Fig 1:**
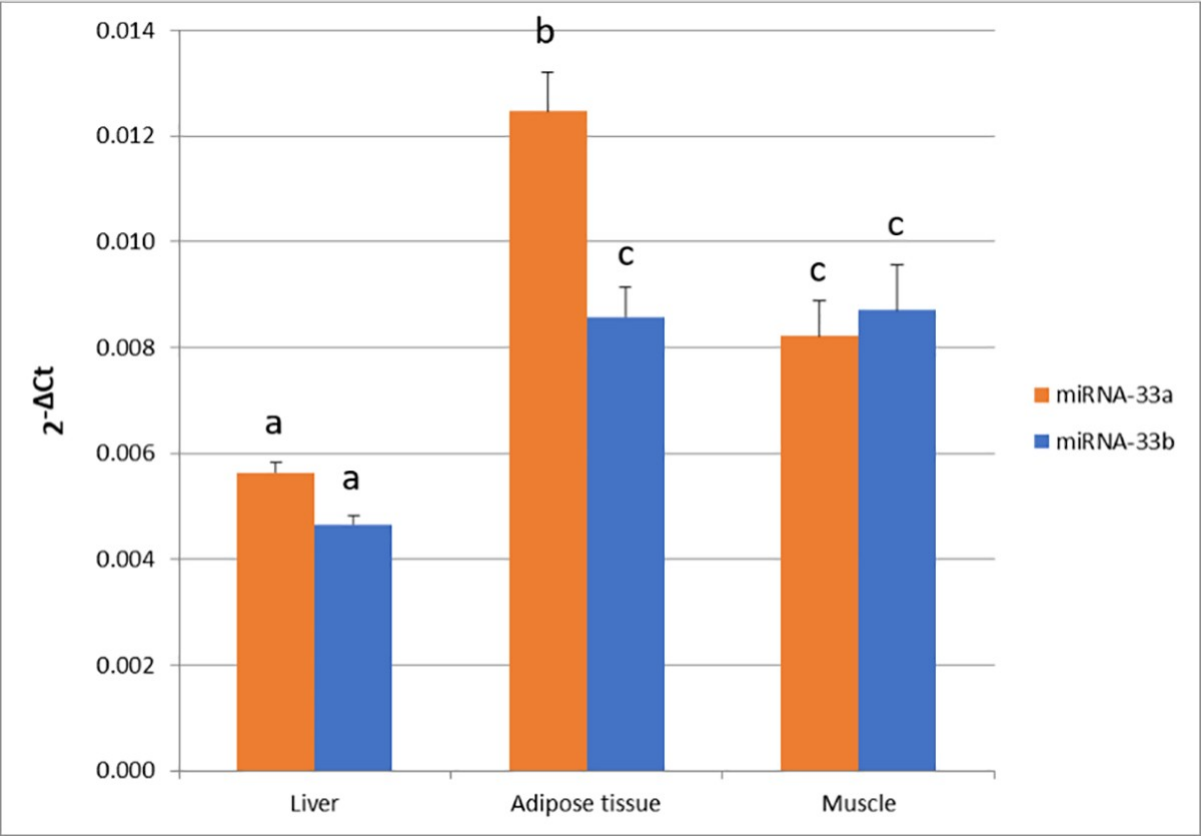
miR-33a and miR-33b expression in liver, adipose tissue and muscle. Data represents 2^-ΔCt^ mean ± standard error of the mean (SEM). Different superscript letters (a, b, and c) indicate significant differences between values (p-value < 0.05) according to the Tukey’s HSD test.

In the current study, miR-33a and miR-33b expression quantification was performed in liver, adipose tissue and muscle of 42 pigs (Fig 1).

**Fig 2:**
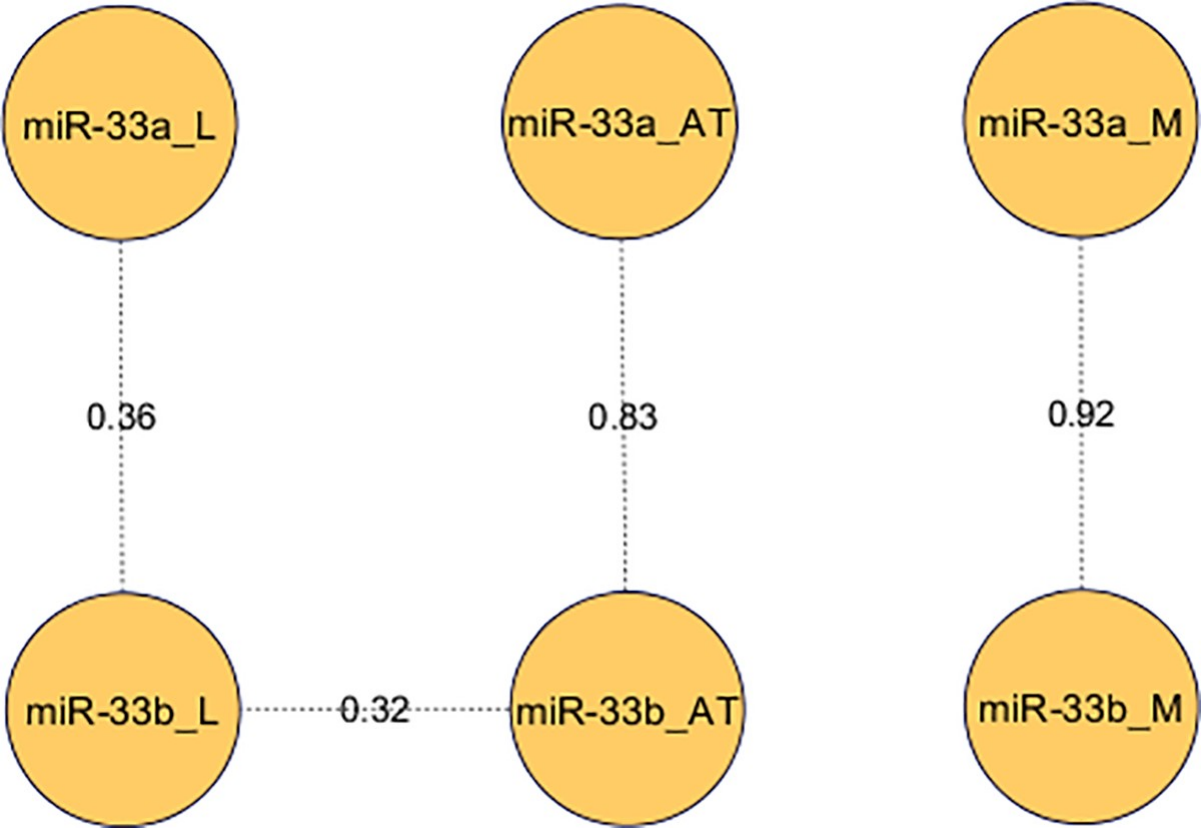
Pearson correlations between miR-33a and miR-33b expression in three tissues. Correlations between miR-33a/b expression in liver (L), adipose tissue (AT) and *longissimus dorsi* muscle (M) were calculated and only significant correlations were represented.

The highest level of miR-33a expression was observed in adipose tissue ($\bar{x}$ = 1.2×10^−02^ ± 1×10^−03^), followed by muscle ($\bar{x}$ = 8×10^−03^ ± 1×10^−03^) and liver ($\bar{x}$ = 6×10^−03^ ± 2×10^−04^). By contrast, miR-33b showed a higher expression in muscle ($\bar{x}$ = 9×10^−02^ ± 1×10^−03^) and adipose tissue ($\bar{x}$ = 9×10^−02^ ± 1×10^−03^) in comparison to liver ($\bar{x}$ = 5×10^−03^ ± 1×10^−04^). Between the two miR-33 genes, miR-33a presented a higher expression than miR-33b in adipose tissue (*p*-value = 1.16×10^−04^). Correlations between miR-33a and miR-33b expression among tissues were calculated (Fig 2), showing a high correlation in muscle (r = 0.92, *p*-value = 2.76×10^−16^) and adipose tissue (r = 0.83, *p*-value = 9.60×10^−11^). Conversely, a lower correlation between miR-33a and miR-33b was observed in liver (r = 0.36, *p*-value = 2.25×10^−02^). Furthermore, correlations among tissues were only significant for liver and adipose tissue miR-33b expressions (r = 0.32, *p*-value = 4.51×10^−02^).

### miR-33a/b expression correlations with *SREBF2* and *SREBF1* respectively

It is well-known that both miR-33a and miR-33b are located in intronic regions of *SREBF2* and *SREBF1* genes, respectively [11, 14, 16, 32]. In order to study if both miR-33a and miR-33b are co-transcribed with their host genes, correlations among their expression levels in the three tissues were calculated and reported in S5 Table. While no significant correlations were found between miR-33b and SREBF1 expression in any tissue, a significant positive correlation between the expression of miR-33a and SREBF2 in liver was found (r = 0.5, p-value = 1.12×10–03).

### Association among the expression levels of miR-33 and target genes

Considering the relevant role that miR-33 members play in lipid and cholesterol metabolism, we wanted to study the association between expression levels of lipid-related genes and miR-33a/b.

To this purpose, previously published mRNA expression levels of 84 lipid-related genes, which were quantified in liver, adipose tissue and/or muscle by qPCR in a set of animals including the 42 animals of the present work [23–25], were used to identify potential binding sites for miR-33. These genes cover different biological functions related to lipid metabolism such as lipolysis and lipogenesis, cholesterol, lipid storage, and transcriptional regulation and control.

The porcine 3’UTRs sequences of the 84 lipid-related genes were downloaded from the Ensembl database and searched for homology with the 7mer seed miR-33 sequence using the Seqkit tool [27]. Fifteen genes containing the 7mer seed miRNA sequence in their 3’UTR were found (Table 1).

**Table 1:** Genes with the 7mer seed miR-33 sequence in their 3’UTR and tissues.

Gene	Tissues
*ACSM5*	Liver, adipose tissue and muscle
*ADIPOQ*	Adipose tissue
*CPT1A*	Liver
*CROT*	Liver, adipose tissue and muscle
*HNF4A*	Liver
*LIPC*	Liver and adipose tissue
*MGLL*	Muscle and adipose tissue
*MLXIPL*	Liver, adipose tissue and muscle
*NCOA1*	Muscle
*NR1H3*	Liver and adipose tissue
*PPARGC1A*	Liver and muscle
*PRKAA1*	Muscle
*SETD7*	Muscle
*SCAP*	Adipose tissue
*USF1*	Liver and adipose tissue

In addition, the 3’UTR target sites conservation between human and pig was evaluated *in silico* using the TargetScan algorithm. The *CPT1A*, *CROT*, *LIPC*, *NCOA1*, *PRKAA1*, and *SETD7* predicted miR-33 target sites were highly conserved across species and showed a context++ score above the 70% percentile, indicating that they are considered cross-validated with confidence and with a high probability of being biologically functional [28].

Low to moderate significant correlations were found among miR-33a and miR-33b and the fifteen target genes containing the 7mer seed miRNA sequence (Table 1) in the three tissues (S6 Table). It is relevant to highlight the negative correlation observed between miR-33b and *CPT1A* expression in liver although it does not reach statistical significance (*p*-value = 0.086). Furthermore, positive correlations were observed between the expression of most of the genes and miR-33a/b, although statistically significant correlations were only obtained between miR-33a and both *PPARGC1A* and *USF1* expression values in liver (*p*-value < 0.05).

### Association between miR-33a and miR-33b expression and fatty acid composition

The association between miR-33a/b expression measured in the three tissues and FA composition measured in backfat adipose tissue and muscle was studied by Pearson’s correlation. While no significant correlations were found between miR-33a/b expression and FA composition measured in muscle, significant and suggestive correlations were found between miR-33a/b expression measured in liver and adipose tissue and FA composition measured in adipose tissue (Table 2). Specifically, liver miR-33a expression was positively correlated with SFA total content, and negatively correlated with linoleic (C18:2(n-6)) and eicosatrienoic (C20:3(n-6)) FA abundances, as well as PUFA total content in adipose tissue. In addition, liver miR-33b expression showed positive correlations with myristic (C14:0) and palmitic (C16:0) FA abundances, and a negative correlation with eicosatrienoic (C20:3(n-6)) FA abundance in adipose tissue. The expression of both miR-33a/b in adipose tissue was positively correlated with the levels of stearic (C18:0) FA and SFA total content, while negative correlations were found with the PUFA total content, along with linoleic (C18:2(n-6)) FA content. Adipose tissue miR-33a expression was also negatively correlated with eicosatrienoic (C20:3(n-6)) FA levels.

**Table 2:** Summary of correlation values between miR-33a/b and FA composition.

	Liver	
FA	miR-33a	miR-33b
C14:0	0.18 (2.60E-01)	0.38 (1.60E-02)
C16:0	0.25 (1.22E-01)	0.34 (3.70E-02)
SFA	0.36 (2.44E-02)	0.20 (2.34E-01)
C18:2(n-6)	-0.40 (1.15E-02)	-0.30 (6.65E-02)
C20:3(n-6)	-0.35 (2.66E-02)	-0.33 (3.91E-02)
PUFA	-0.38 (1.63E-02)	-0.29 (6.94E-02)
	Adipose tissue	
FA	miR-33a	miR-33b
C18:0	0.35 (2.82E-02)	0.35 (3.12E-02)
SFA	0.32 (4.41E-02)	0.42 (7.93E-03)*
C18:2(n-6)	-0.41 (9.90E-03)**	-0.49 (2.03E-03)*
C20:3(n-6)	-0.34 (3.42E-02)	-0.29 (8.22E-02)
PUFA	-0.40 (1.09E-02)**	-0.48 (2.16E-03)*

miRNAs were measured in liver and adipose tissue and FA composition was measured in backfat adipose tissue. *P*-values are indicated in brackets and * and ** means statistically significant (FDR-based *q*-value < 0.05) or suggestive (FDR-based *q*-value < 0.1), respectively.

## Discussion

Since the miR-33 family has a relevant role in the regulation of genes involved in lipid metabolism pathways, in the current work, the expression of miR-33a and miR-33b in liver, adipose tissue and muscle, and its correlation with both *in-silico* predicted target lipid-related genes and FA composition traits were studied.

Several studies in humans and mice have reported that miR-33a and miR-33b are co-transcribed with their host genes, *SREBF2* and *SREBF1*, respectively [11, 14, 32–34]. However, a low correlation between miR-33b and *SREBF1* gene expression has been reported in adipose tissue of pigs [16] and members of the miR-33 family are not co-regulated with their host genes in most tissues of chickens [35]. Accordingly, in the present study, no significant correlation was observed between miR-33b and *SREBF1* gene expression in any tissue. On the contrary, miR-33a and *SREBF2* gene expression in liver showed a positive correlation (r = 0.5). Overall, these results suggest that both miRNAs are transcribed in different ways.

Additionally, the analysis of miR-33a and miR-33b expression in liver, adipose tissue and muscle revealed different expression patterns among tissues for both miRNAs. Similar results have been also reported in humans with different levels of miR-33a and miR33-b expression depending on tissue [36], which suggests that different tissue-specific mechanisms are regulating the expression of miR-33a/b. Conversely, high correlations between miR-33a and miR-33b expression levels (r > 0.8) were obtained within the muscle and the adipose tissues, suggesting a similar regulation in the expression of both miRNAs in these tissues. In fact, taking into account that both miR-33a/b have the same seed sequence, we cannot discard that both miR-33a/b play a similar function in these tissues. However, different expression levels between miR-33a and miR-33b in adipose tissue were found. To the best of our knowledge, there are no published works regarding the role of miR-33a in the adipose tissue of pigs. A study published in humans determined that miR-33a was constitutively expressed while miR-33b expression increased during adipocyte differentiation [37]. Conversely, transfection of miR-33b in porcine subcutaneous preadipocytes downregulates adipose differentiation and lipid accumulation [16]. Thus, further studies are necessary to better understand the role of miR33a in pig adipose tissue and determine if both miR-33a/b have different regulatory functions in this tissue.

A different expression pattern was observed for both miR-33a/b in liver, where the lowest expression levels and correlation values between miR-33a and miR-33b (r = 0.36) were obtained. It has been reported that miR-33a and miR-33b work in collaboration with their host genes regulating lipid metabolism in liver, and while miR-33a participates in the transcriptional control of genes involved in cholesterol pathways [12, 14, 15, 32, 33], miR-33b was related with fatty acid oxidation and insulin signalling pathway [11, 34]. In pigs, liver plays an important role in *de novo* cholesterol synthesis, lipogenesis and fatty acid oxidation [4–6, 38, 39]. In line with the low correlation values observed between both miR-33a/b in liver, miR-33b tended to be higher negatively correlated with *CPT1A* expression levels than miR-33a. Therefore, we could hypothesize that both miR-33a/b plays a different regulatory role in liver, with miR-33b being involved in FA β-oxidation. This is also supported by the positive correlations between the expression of miR-33a and two genes (*PPARGC1A* and *USF1*) found in liver, because these two genes are transcription factors involved in the regulation of several genes of fatty acid metabolism [40–42].

**Fig 3:**
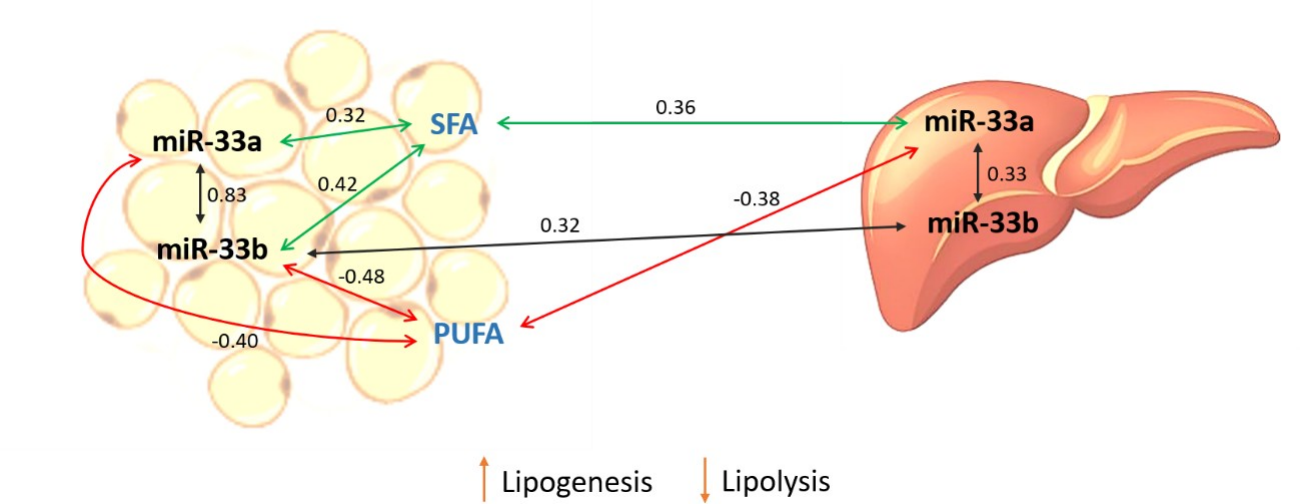
Schematic representation of correlation results. Correlations were calculated between miR-33a/b expression measured in liver and adipose tissue and FAs measured in adipose tissue. Green and red lines represent positive and negative correlations respectively. Black lines indicate correlations of the expression of miR-33a and miR-33b within and between tissues.

In general, suggestive significant positive correlations between miR-33a/b expressions in either liver and adipose tissue and SFAs, whereas suggestive negative correlations with PUFAs were observed (Fig 3). It is well-known the role of PUFA in the expression regulation of genes implicated in FA β-oxidation, adipogenesis, and lipogenesis *de novo* [43, 44]. Previous studies of our group reported that BC1_LD animals with a higher intramuscular content of PUFA increased the expression of genes involved in the fatty acid oxidation and cholesterol homeostasis in liver and inhibited lipogenesis pathways in liver and adipose tissue [45, 46].

In the current study, liver miR-33a was negatively correlated with adipose tissue PUFA content (r = -0.38). Taking into account that liver is the main organ implicated in the cholesterol metabolism and secretion of very low-density proteins, we can hypothesize that animals with high expression levels of miR-33a increase the cholesterogenesis pathway, and therefore the transport of different FAs, notably to the adipose tissue in order to accumulate triglycerides, increasing the content of SFA which are used in the lipogenesis process.

Supporting this hypothesis, in adipose tissue both miR-33a/b were negatively correlated with the total PUFA content, although only miR-33b reached the threshold for significance. Porcine miR-33b has been reported to play an important role in adipogenesis and lipogenesis in adipose tissue, as well as in the control of triglyceride levels [16]. Hence, an interaction between cholesterol and lipogenesis pathways may explain the correlation between the miR-33 expression in liver and adipose tissue and the FA composition measured in the adipose tissue.

Despite that, we cannot determine the cause-effect direction of the interaction between the miR-33a/b and the PUFA content, and further functional analyses are needed to better understand the role of miR33 members in the determination of FA composition in adipose tissue or vice versa. This is of great interest because fatty acids in both muscle and adipose tissue are determinant of meat quality and its nutritional values. In detail, SFA consumption has been related to modern human diseases such as obesity, cancer and cardiovascular diseases, while PUFAs are directly related with a decrease of meat quality and a reduction of total cholesterol concentration [1, 47].

## Conclusions

Our study manifested that miR-33a and miR-33b were transcribed in a different manner and the miR-33a/b expression regulatory mechanisms were different according to tissue. miR-33a and miR-33b expression levels presented high correlations in adipose tissue and muscle which may indicate a similar regulation of their expression in these two tissues. Conversely, in liver, the different expression pattern and low expression correlation between miR-33a and miR-33b found, together with the negative correlation between miR-33b and *CPT1A* expression and positive correlations between miR-33a and *PPARGC1A* and *USF1* expression, indicates that both miRNAs have different functions and miR-33b may be involved in FA-β-oxidation. However, further functional validation studies are needed to demonstrate the miRNA regulation of *in-silico* target genes involved in lipid metabolism. Finally, positive and negative correlations between miR-33a/b expression and SFA and PUFA content, respectively, were identified which suggested a possible role of miR-33 family in the determination of FA composition in adipose tissue.

## Supporting information

S1 Table Descriptive statistics including mean and SD of intramuscular fat and backfat Fatty Acid (FA) composition and FA indices in the 42 animals analysed. (XLSX)

S2 Table Primers used for *SREBF2*, *ACTB* and *TBP* gene expression quantification by qPCR. (XLSX)

S3 Table miR-33a, miR-33b and *SREBF2* gene expression data in liver (LV), adipose tissue (BF), and muscle (LD). (XLSX)

S4 Table List of genes analysed by qPCR in each tissue (Puig-Oliveras et al., 2016; Ballester et al., 2017; Revilla et al., 2018). (XLSX)

S5 Table Pearson’s correlation values between miR-33a and *SREBF2* gene expression, and between miR-33b and *SREBF1* gene expression. (XLSX)

S6 Table Pearson’s correlations between miR-33a/b and mRNA target genes expression values. *P*-values are indicated in brackets and * means statistically significant (*p*-value < 0.05).
(XLSX)
